# Genetic modifiers of p53: opportunities for breast cancer therapies

**DOI:** 10.18632/oncotarget.28387

**Published:** 2023-03-24

**Authors:** Prabin Dhangada Majhi, Aman Sharma, D. Joseph Jerry

**Keywords:** p53, Li-Fraumeni syndrome, genetic modifiers, breast cancer, DNA repair

## TP53 mutations: a critical breach in the barriers to breast cancer

**Figure 1:**
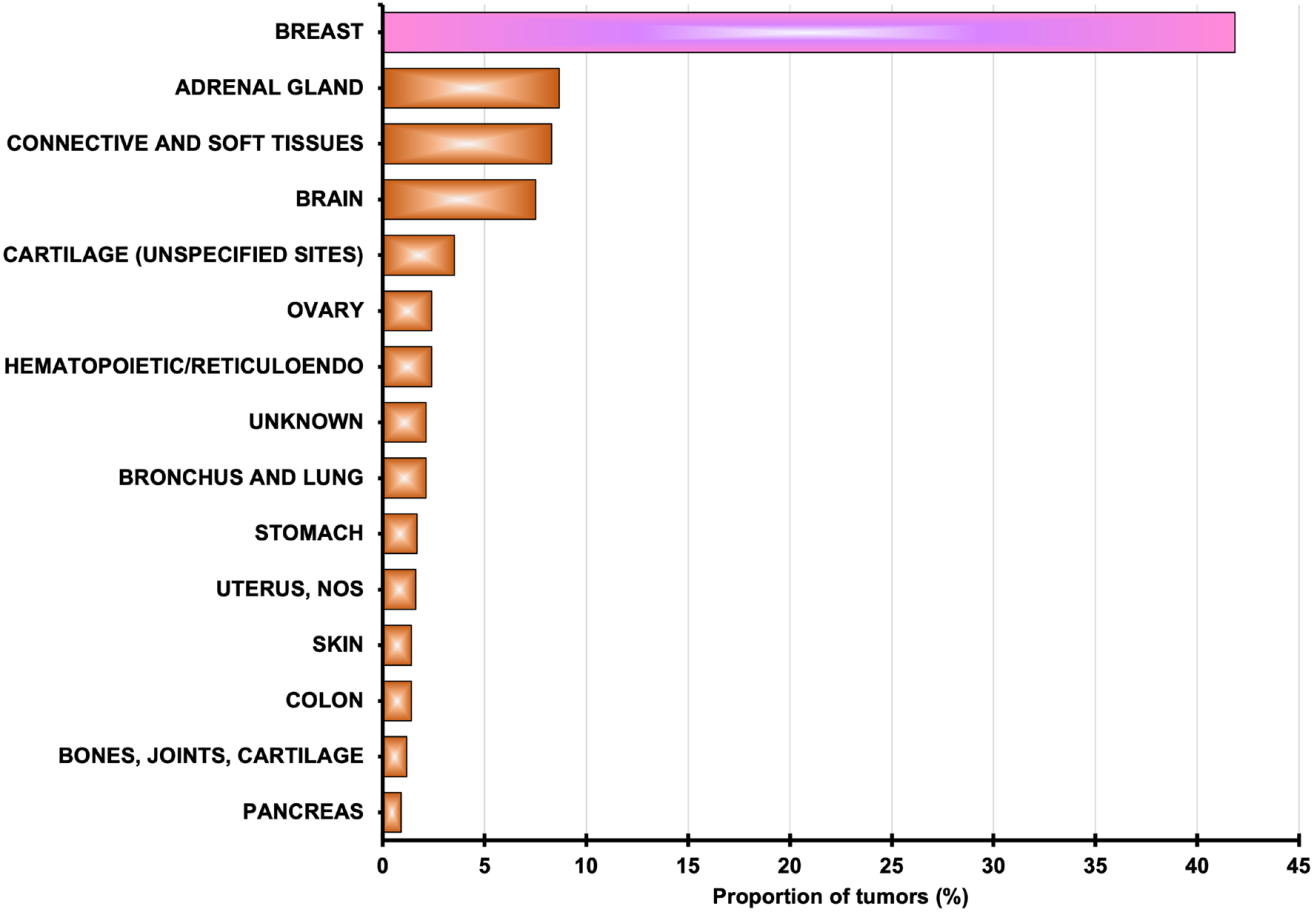
Tumor spectrum among women with inherited mutations in *TP53*. Data from the *TP53* Database, release R20 [3].

Each day our cells encounter a wide range of genomic damage and the p53 protein arbitrates decisions of cell cycle arrest to allow repair of DNA or promote elimination of cells with malignant potential through apoptosis. The prevalence of *TP53* mutations in nearly all tumors emphasizes its role as a formidable barrier that must be breached to allow oncogenic transformation. Inherited mutations in *TP53* are also the primary genetic lesions found in Li-Fraumeni Syndrome (LFS), a familial cancer predisposition characterized by tumors in many tissues [1, 2]. However, tissues are not all equally vulnerable to disruptions in p53 function. Among women with inherited mutations in *TP53*, breast cancer is by far the most common tumor (Figure 1) [3]. Somatic mutations in *TP53* are also prevalent in sporadic breast cancers, especially in the triple-negative subtype [4]. The proportion rises to nearly 50% of breast cancers that exhibit impaired function of the p53 pathway based on gene expression signatures as a surrogate biomarker of p53 activity [5–7]. Therefore, the breast epithelium appears to be uniquely sensitive to alterations in p53 function.

**Figure 2:**
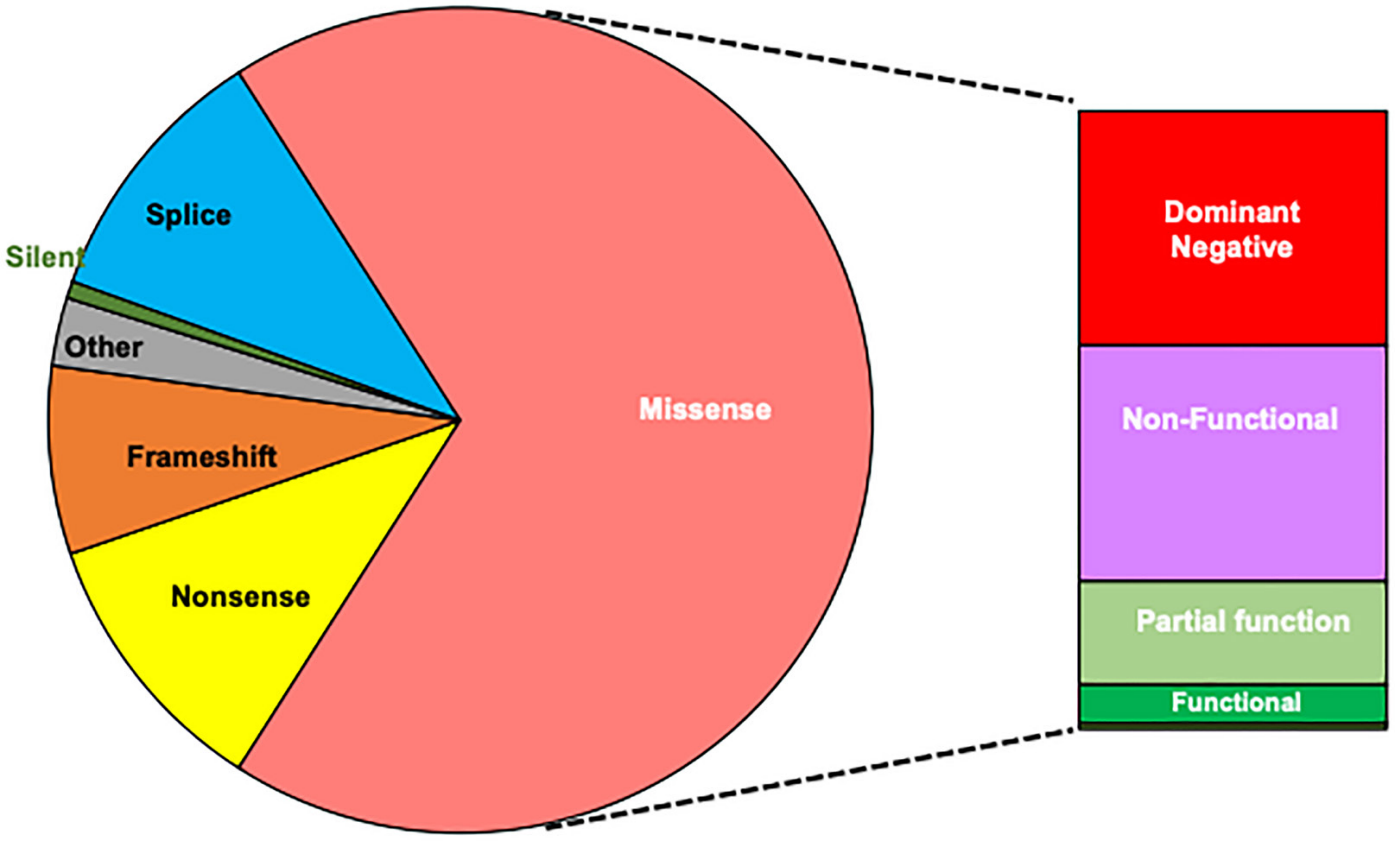
Distribution of germline *TP53* mutations among women developing breast cancer. Missense mutations are the most common. Hotspot mutations in R175, G245, R248 and R273 associated with dominant-negative activities are found in 26%; partial or complete loss of function in 37% while 5% retain transactivation activities. The remaining 31% of mutations result in truncated proteins with loss of function with 0.7% designated as silent. *TP53* mutation database, release 18 [8].

The pattern of cancer-associated mutations in *TP53* is distinct among tumor suppressor genes as missense mutations are the most common. Missense mutations are distributed throughout p53 leaving the protein-coding region intact but its function altered. This pattern is also found in women with inherited mutations in *TP53* who develop breast cancers (Figure 2) [8]. Missense mutations affecting codons R175, G245, R248 and R273 within the DNA binding domain account for 26% of the total and have been associated with dominant-negative activities. The majority of the remaining 37% of missense mutations exhibit partial or complete loss of function based on transactivation criteria [8]. Nonsense, frameshift, splice mutations and deletions make up the remaining 31% and also cause loss of function in most cases. Thus, the mutational spectrum is similar to that found in other cancers and cannot account for the prevalence of breast cancer in LFS. As loss-of-function is the most common consequence of mutations, it suggests that integrity of the p53 pathway is especially critical in LFS-related breast cancers.

## Genetic modifiers: a supporting cast in tumor suppression

The consequences of inherited mutations in *TP53* vary widely among individuals as well as among tissues. The classic definition of LFS was familial clustering of soft tissue sarcomas before age 45 together with another first-degree relative with any cancer prior to 45 years or sarcoma at any age [9]. In 1990, LFS was linked to inheritance of germline pathogenic variants in the *TP53* gene in 5 families [10]. Since that time the clinical definition of LFS has been expanded to include diverse tumors and clinical features to guide referrals for testing for germline mutations in *TP53* [11]. Penetrance of LFS is much more variable than previously recognized with approximately 20% of *TP53* mutations carriers being detected in individuals outside of a familial cancer context [1]. The time of onset and tissues where tumors arise vary greatly with children developing tumors early in life only to find that the parent also carried the mutation in *TP53* but remained unaffected. These observations suggested genetic anticipation in which mutations accumulate progressively among generations causing earlier onset and increased severity of cancers. However, whole genome sequencing failed to identify evidence of increased copy number variants among generations [12]. There is also no evidence of genetic anticipation in strains of rodents carrying mutations in the p53 gene (designated *Trp53*). The variable penetrance of disease among individuals with inherited mutations in *TP53* suggests that other factors can modify the tumor spectrum and latency. A functional variant in miR-605 was implicated as a genetic modifier [13, 14]. A polymorphism in the promoter of *MDM2* at SNP309 (T>G) was shown to increase levels of *MDM2* expression leading to diminished function of p53 [15]. Additional genetic polymorphisms implicated in modifying p53 activity were reviewed recently [1, 16]. However, the extent to which these account for the variation in latency of tumors and tissues affected remains to be established.

Genetic studies in rodents provide clearer evidence of genetic modifiers contributing to the variable penetrance of both gain-of-function and loss-of-function mutations in *Trp53*. C57BL/6 mice with the R172H mutation (equivalent to R175H in human p53) were crossed with 129Sl, A/J, BALB/cByJ, C3H, DBA, NOD and SWR recombinant inbred strains of mice [17]. The F1 mice heterozygous for the R172H mutation in *Trp53* exhibited striking differences in the latency of tumors with 50% incidence at 13 months in crosses with the A/J strain compared to 20 months for crosses with the NOD strain. The tissues affected also differed. While lymphomas are the most common tumor overall, the frequency was 30% of the [C57BL/6xSWR]F1 mice compared to 4% in [C57BL/6xBALB/c]F1 mice heterozygous for R172H. The incidence of adenocarcinomas was greatest in the [C57BL/6xBALB/c]F1 mice and lowest in the [C57BL/6x129SL]F1 (13% vs. 2% respectively). Incidence of tumors is also significantly higher in the [C57BL/6xBALB/c]F1 males compared to females but this was not observed in other crosses. Therefore, genetic polymorphisms differing among these strains alter the latency, tissues affected and exhibit sexual dimorphism of tumors in mice carrying the heterozygous mutation of R172H. The results demonstrate the presence of dominant alleles that can potentiate the penetrance of tumors as well as mitigate the effects of the R172H mutation in mice. Therefore, p53 acts in concert with other genes that alter the penetrance of tumor phenotypes and the modifiers differ among tissues.

**Figure 3:**
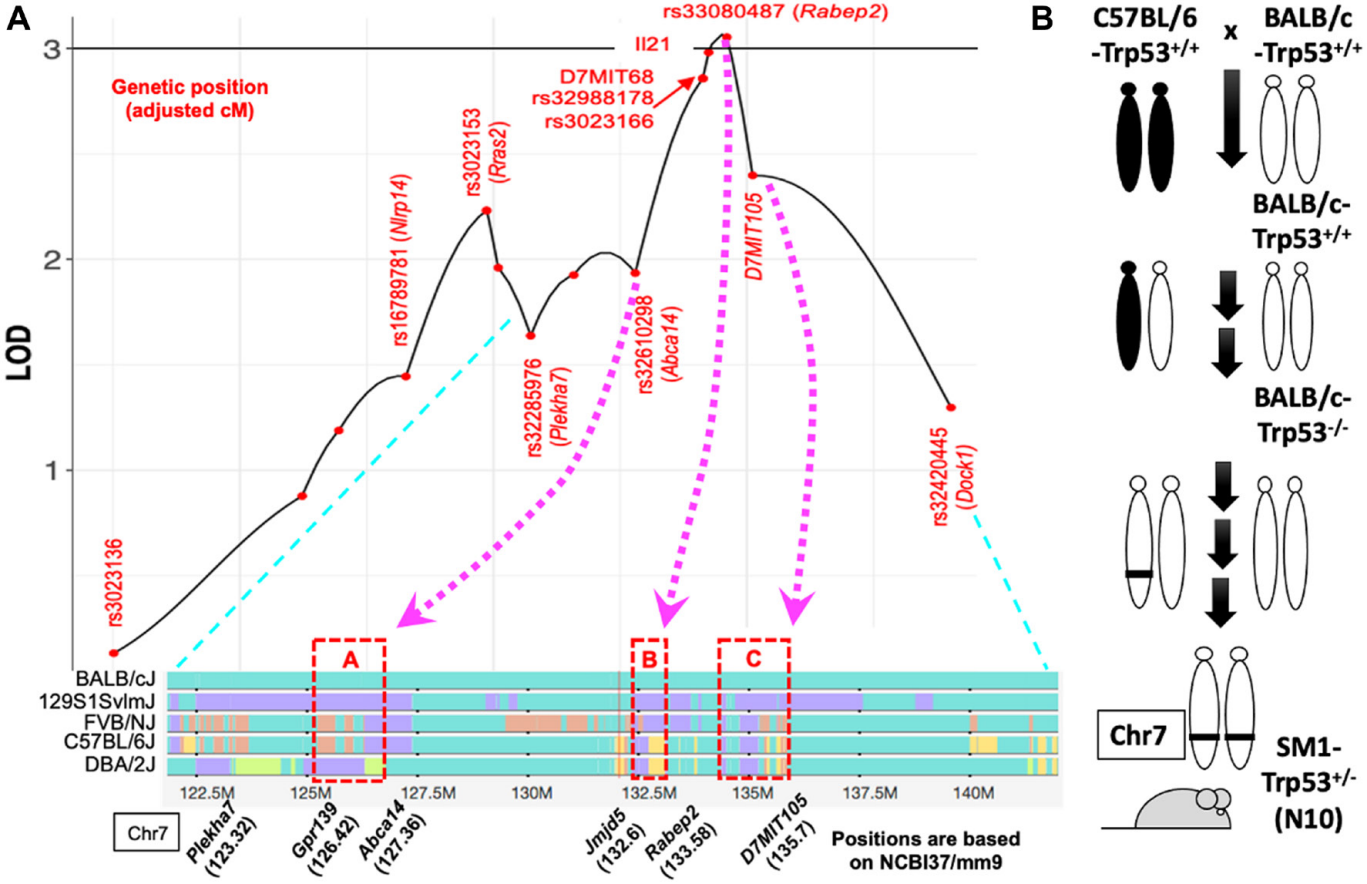
Genetic linkage and haplotypes associated with mammary tumors in Trp53+/− mice. (**A**) Linkage data and haplotype blocks were reported in Majhi et al., 2021 [30]. (**B**) C57BL/6 alleles on chromosome 7 (Chr7:121,851,009-132,031,773; NCBI37/mm9) were introgressed into the BALB/c background to create congenic SM1 mice.

Mice heterozygous for null mutations in *Trp53* also develop spontaneous tumors in a range of tissues. The tumor spectrum for *Trp53*+/− mice has been reported for C57BL/6, BALB/c, FVB, DBA/2, C3H/H3N and 129Sv strains [18–25]. Among these strains, BALB/c females are uniquely susceptible to mammary tumors despite similar prevalence of other tumor types [18]. Therefore, the genetic modifiers in BALB/c mice appear to selectively increase the risk of mammary tumors in females. Although mammary tumors are rare in C57BL/6-*Trp53*+/− mice, crossing with BALB/c increased the incidence of mammary tumors which was further increased when these mice were backcrossed to BALB/c (N2 mice). This demonstrates the polygenic nature of susceptibility and resistance to mammary tumors in mice as well as the presence of both dominant- and recessive-acting alleles [20]. The strains also differ in the frequency of loss of heterozygosity at *Trp53* in the tumors suggesting differences in DNA damage and repair mechanisms in BALB/c and C57BL/6 strains may play a role [26]. Genetic mapping identified multiple loci with a major locus linked to mouse chromosome 7 [21, 27]. Analysis of haplotype blocks across the locus on chromosome 7 (designated *Suprmam1*) identified 3 regions where BALB/c polymorphisms differ from the 4 strains with very low incidence of mammary tumors (Figure 3A). Although coding polymorphisms are present among genes within the haplotype blocks, none appeared to alter the function or expression of the genes.

Among the 12 major genes associated with inherited risk of breast cancer in humans [28], 9 disrupt the recognition of DNA double-strand breaks (DSBs) or homology-directed repair. Canonical homologous recombination allows precise repair of DSBs, but other homology-directed mechanisms are considered error-prone. These include single-strand annealing (SSA), microhomology-mediated end-joining (MMEJ) and alternative end-joining (AltEJ). Thus, a dynamic balance of error-free and error-prone DSB repair pathways exists within cells. In cells from BALB/c-*Trp53*+/− mice, repair of DSBs by low fidelity SSA/AltEJ was 3-fold greater than in C57BL/6-*Trp53*+/− cells [29]. It is important to note that the differences in DSB repair pathways were conditional upon the p53 status as it was not detected in *Trp53*+/+ cells from these strains consistent with haploinsufficiency in the phenotypes.

To determine whether the *Suprmam1* locus was involved, F1 mice were backcrossed to introgress a 20 Mb interval of C57BL/6 alleles onto the BALB/c genetic background (Figure 3B). The studies replicated the ~3-fold greater use of low fidelity SSA/AltEJ in cells from BALB/c-*Trp53*+/− compared to the C57BL/6-*Trp53*+/− strain. In SM1 mice, the C57BL/6 alleles in *Suprmam1* were sufficient to revert the DSB repair to that of the C57BL/6 parent [30]. Therefore, BALB/c and C57BL/6 strains differ in the balance of pathways used to repair DSBs and are genetically linked to the *Suprmam1* locus. These studies demonstrate the powerful effect of genetic modifiers to compensate for deficiencies in p53 and that they act in a tissue-specific manner to alter susceptibility to mammary tumors but not to other tumor types.

## Genetic modifiers as targets for therapies

The p53 checkpoint is a potent inhibitor of malignancy. Mice bearing loss-of-function mutations develop tumors, but upon genetic restoration of the *Trp53* gene, the tumors regress rapidly [31–33]. Therefore, drugs that restore wild-type function to the mutant proteins have been a focus of many efforts [34–36]. Few of the strategies have reached clinical trials and mutant p53 proteins remain largely “undruggable”. Another hurdle is that the presence of pathogenic mutations in *TP53* do not predict patient outcomes, and therefore, is not used routinely to guide clinical care for breast cancer patients. This is due to the lack of consistent relationships between *TP53* status and outcomes among clinical trials. Stratification of METABRIC data showed no difference in overall survival between breast tumors with wild-type or mutant p53 among women treated with chemotherapies [37]. However, the authors also noted that studies have been limited by sizes of cohorts, methods for determining p53 status and confounding among treatments. Therefore, the utility of p53 as a clinical biomarker remains to be fully tested.

**Figure 4:**
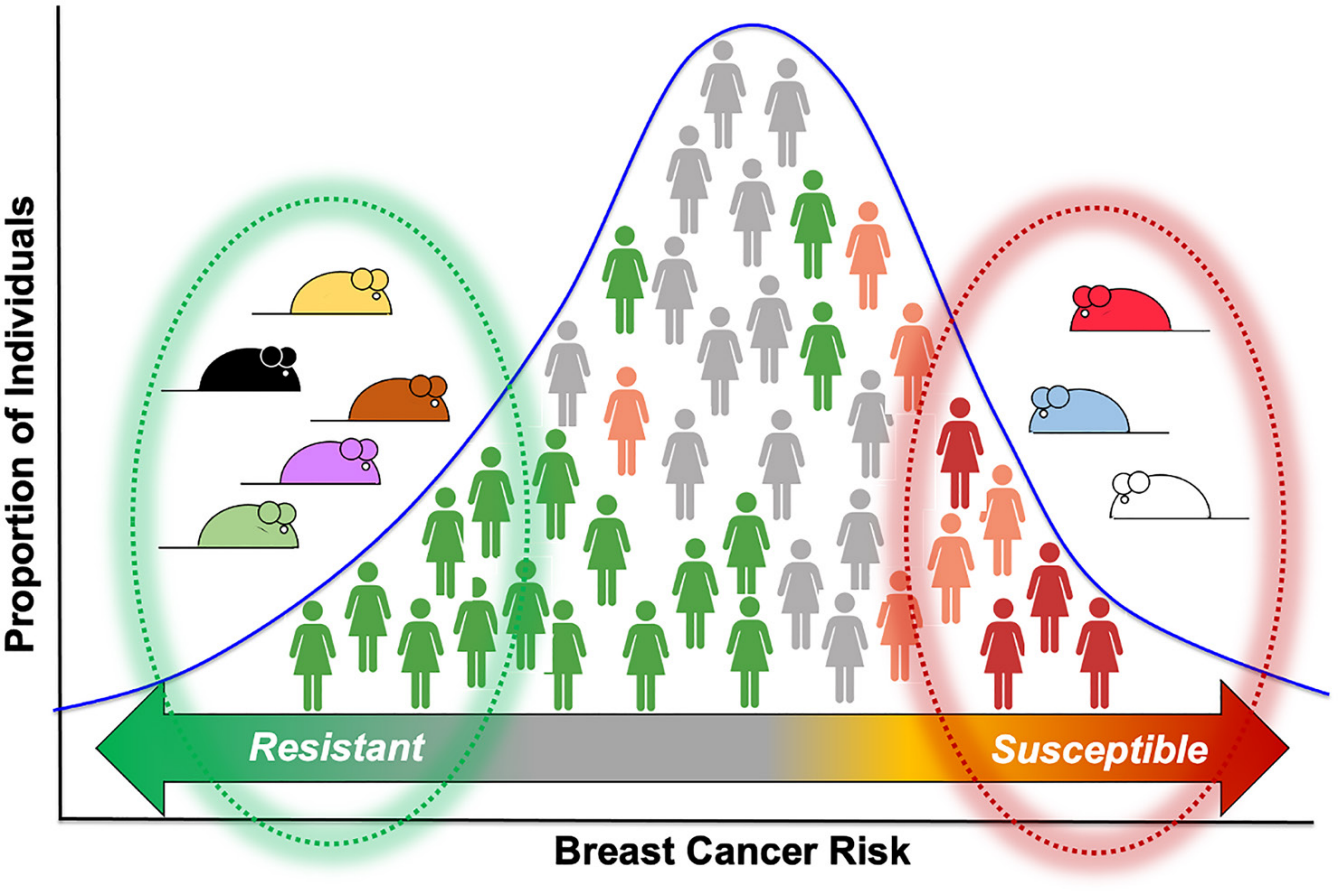
Loss of p53 predisposes to mammary tumors in mice and in women, but penetrance varies among individuals. Genetic polymorphisms associated with breast cancer risk in humans may act on these pathways to compensate for or mitigate the effect of p53 loss offering targets for therapies.

Effects of genetic modifiers can also influence the impact of *TP53* mutations on clinical outcomes. Genetic polymorphisms in mice provide dramatic examples of the effects of genetic modifiers contributing to the variable penetrance of *Trp53* mutations. While complicating our ability to predict the consequences of mutations in *TP53* for individuals, the genetic modifiers reveal the presence of cellular mechanisms that can compensate for the disruptions of p53 function and prevent progression of tumors. Strains of mice differing in their sensitivity to mutations in *Trp53* provide a genetic resource with which to identify the functional polymorphisms and pathways that confer resistance to tumors. The genetic modifiers in mice can also guide the search for genetic polymorphisms affecting these pathways in humans (Figure 4). Genome-wide association studies (GWAS) have identified over 300 polymorphisms that contribute to breast cancer risk [38–41]. These provide a rich resource of candidate polymorphisms that may modify the consequences of mutations in *TP53*. Pathways that compensate for disruption of p53 function offer potent new targets for therapies to treat triple-negative breast cancers which are enriched for mutations in *TP53* and preventive therapies for those with inherited mutations. Identification of genetic pathways that collaborate with disruption of p53 function will also provide valuable guidance in predicting the behavior of tumors bearing mutations in *TP53*. Realizing the opportunities for drugging the cast of collaborators will rely on redoubling efforts to identify the mechanisms underlying the genetic modifiers of p53.
